# Angiotensin II-derived constrained peptides with antiplasmodial activity and suppressed vasoconstriction

**DOI:** 10.1038/s41598-017-14642-z

**Published:** 2017-10-30

**Authors:** Adriana Farias Silva, Marcelo Der Torossian Torres, Leandro Souza Silva, Flavio Lopes Alves, Ana Acácia de Sá Pinheiro, Antonio Miranda, Margareth Lara Capurro, Cesar de la Fuente-Nunez, Vani Xavier Oliveira

**Affiliations:** 1 Centro de Ciências Naturais e Humanas, Universidade Federal do ABC, Santo André, SP Brazil; 2 Synthetic Biology Group, MIT Synthetic Biology Center, Massachusetts Institute of Technology, Cambridge, Massachusetts, USA; 3 Research Laboratory of Electronics, Massachusetts Institute of Technology, Cambridge, Massachusetts, USA; 4 Department of Biological Engineering, and Department of Electrical Engineering and Computer Science, Massachusetts Institute of Technology, Cambridge, Massachusetts, USA; 5 Broad Institute of MIT and Harvard, Cambridge, Massachusetts, USA; 6 The Center for Microbiome Informatics and Therapeutics, Cambridge, Massachusetts, USA; 7 Instituto de Biofísica Carlos Chagas, Universidade Federal do Rio de Janeiro, Rio de Janeiro, RJ Brazil; 8 Departamento de Biofísica, Universidade Federal de São Paulo, São Paulo, Brazil; 9 Departamento de Parasitologia, Instituto de Ciências Biomédicas II, Universidade de São Paulo, São Paulo, SP Brazil

## Abstract

Angiotensin II (Ang II) is a natural mammalian hormone that has been described to exhibit antiplasmodial activity therefore constituting a promising alternative for the treatment of malaria. Despite its promise, the development of Ang II as an antimalarial is limited by its potent induction of vasoconstriction and its rapid degradation within minutes. Here, we used peptide design to perform targeted chemical modifications to Ang II to generate conformationally restricted (disulfide-crosslinked) peptide derivatives with suppressed vasoconstrictor activity and increased stability. Designed constrained peptides were synthesized chemically and then tested for antiplasmodial activity. Two lead constrained peptides were identified (i.e., peptides 1 and 2), each composed of 10 amino acid residues. These peptides exhibited very promising activity in both our *Plasmodium gallinaceum* (>80%) and *Plasmodium falciparum* (>40%) models, an activity that was equivalent to that of Ang II, and led to complete suppression of vasoconstriction. In addition, peptide 5 exhibited selective activity towards the pre-erythrocytic stage (98% of activity against *P. gallinaceum*), thus suggesting that it may be possible to design peptides that target specific stages of the malaria life cycle. The Ang II derived stable scaffolds presented here may provide the basis for development of a new generation of peptide-based drugs for the treatment of malaria.

## Introduction

Malaria is a mosquito-borne infectious disease affecting humans and other animals caused by parasitic protozoa from the genus *Plasmodium*. Five hundred thousand people die annually from malaria, mostly children under the age of five, with 90% of cases occurring in Sub-Saharan Africa^1,2^. In addition, an estimated 250 million people suffer from malaria each year^1,2^.

Angiotensin II (Ang II) is an octapeptide hormone that plays a central role in cardiovascular homeostasis, acting as a vasoactive agonist that induces contraction of blood vessels. Ang II raises blood pressure via vasoconstriction, sympathetic nervous stimulation, and increased aldosterone biosynthesis and renal actions. This peptide has been shown to exhibit antiplasmodial activity^3–8^, thus representing a promising novel anti-malaria drug. However, Ang II is limited by its rapid degradation (i.e., short half-life)^9^, and its potent vasoconstrictor effect^10^.

Recent efforts have focused on modifying the primary structure of Ang II to generate synthetic variants with conserved antiplasmodial activity but suppressed vasoconstrictor effect^3–8^. For example, Maciel *et al*., synthesized six Ang II analogs, three linear and three restricted peptides, designed via introduction of two amino acid residues (D and K) to the N-terminus. This resulted in the formation of a lactam bridge with a scaffold that increased the potency of the peptide towards parasite lipid membranes^4,11^. In the same study, linear VC-4 (DDRKVYIHPF) and restricted VC-5 (DRDVKYIHPF) peptides presented biological activity (64% and 78%, respectively), but only VC-5 displayed complete depletion of vasoconstrictor effects^4^.

In constrained peptides, the insertion of a lactam bridge at the Ang II N-terminus increased the antiplasmodial activity when compared to peptides that contain the lactam ring within the C-terminus^3^. To understand the effect of ring position, bridgehead elements and ring size of Ang II derivatives, Torres *et al*. further modified the VC-5, VC-17 and VC-19 analogs^6^. New peptides containing i-(i + 2); i-(i + 3) or i-(i + 4) lactam bridge scaffolds were synthesized with E or D and O or K amino acid residues, and D- and L-enantiomers as bridgehead elements. Three of these analogs (i.e., EDRKVYIHPF; EDRVOYIHPF; eDRVOYIHPF) maintained the antiplasmodial activity of Ang II. Almost all the designed peptides tended to adopt a β-turn conformation, in agreement with the conformational tendency of the native molecule, except for eDRVOYIHPF, which tended to adopt an α-helix conformation in the studied solvents^6^.

Silva *et al*.^12^, tested several linear Ang II derivative peptides *in vitro* using both the *P. gallinaceum* sporozoite and the *P. falciparum* blood stage models. Two short peptides presented high antiplasmodial activity: YHPF and VIPF presented 89% and 94% activity against *P. gallinaceum*, respectively, and ~50% activity against erythrocyte invasion by *P. falciparum*. These derivatives exhibited an activity that was equipotent to that of Ang II against Plasmodium sporozoites and repressed pressor activity.

The small linear peptides^12^ and the constrained derivatives previously described^7,8^ were used here as templates for the design of a new class of stable^13^ constrained peptides that retain higher antiplasmodial (~98% inhibition) activity cf. Ang II (~88% inhibition). These constrained peptides suppressed vasoconstriction effects similar to previously described Ang II analogs. Our findings present novel peptides with selective activity against the pre-erythrocytic cycle of malaria and represent promising templates for the prevention of malaria.

## Results

### Peptide design, synthesis and characterization

All peptides were designed, synthesized and evaluated for their potential as novel antiplasmodial drugs. These analogs were purified and characterized as described in the Methods Section, resulting in a chromatographic purity >96% in all cases. Mass characterization by LC/MS-ESI (+) confirmed the chemical identity of peptides and was in agreement with the expected molecular weight theoretical values (Table 1).

**Table 1:** Design of angiotensin II-derived synthetic peptides. Purity of peptides determined by LC/MS is shown, along with their respective antiplasmodial activity and design rationale.

Entry	Sequence	HPLC purity^a^ (%)	Calculed mass^b^ (g mol^−1^)	Observed mass^b^ (g mol^−1^)	Antiplasmodial activity vs *P. gallinaceum* (% fluorescent sporozoites)	Antiplasmodial activity vs *P. falciparum* (new ring formation%)	Design rationale
**Ang II**	DRVYIHPF				88	47	WT peptide
**1**	CDRVYIHPFC	98	1249.6	1250	92	42	Constrained version of Ang II.
**2**	CDRVYHIPFC	98	1249.6	1250	91	42	Residues H and I were reversed, which is expected to increase antiplasmodial activity based on previously described linear variant^12^
**3**	CDRVCYHIPF	98	1249.6	1250	82	11	Designed based on most potent antiplasmodial peptide (CDRVCYIHPF) obtained previously^16^, with an additional IH reverse modification.
**4**	CDRVYPFC	97	999.4	1000	95	9	Hydrophobic patch composed of H and I was deleted to assess its influence on function.
**5**	CRYHIPFC	98	1035.5	1036	98	0	D and V were deleted to optimize interactions between R and Y within the same plane. I and H were reversed to evaluate the effect of new pi-stack interactions in the activity.
**6**	CRYPFC	99	785.3	786	4	29	Most important residues for function within the Ang II sequence were kept to build a minimal peptide with antiplasmodial activity.
**7**	CYHPFC	97	766.3	767	6	17	Only aromatic residues were kept inspired by the linear form (YHPF), which was one of our lead peptides previously described^12^.
**8**	CVIPFC	98	675.3	676	7	3.5	Aliphatic and hydrophobic residue F were kept, based on previous lead linear peptide (VIPF)^12^.

^a^HPLC profiles were obtained under the following conditions: Column Supelcosil C18 (4.6 × 150 mm), 60 Å, 5 μm; Solvent System: A (0.1% TFA/H2O) and B (0.1% TFA in 60% ACN/H2O); Gradient: 5–95% B in 30 min; Flow: 1.0 mL/min; λ = 220 nm; Injection Volume: 50 μL and sample concentration: 1.0 mg/mL. ^b^The masses were determined by LC/ESI–MS using a Micromass instrument (model ZMD) coupled to a Waters Alliance (model 2690) system. Mass measurements were performed in a positive mode with the following parameters: mass range between 300 and 2000 m/z; nitrogen gas flow: 4.1 L/h; capillary: 2.3 kV; cone voltage: 32 V; extractor: 8 V; source heater: 100 °C; solvent heater: 400 °C; ion energy: 1.0 V and multiplier: 800 V.

### Circular Dichroism (CD)

**Figure 1:**
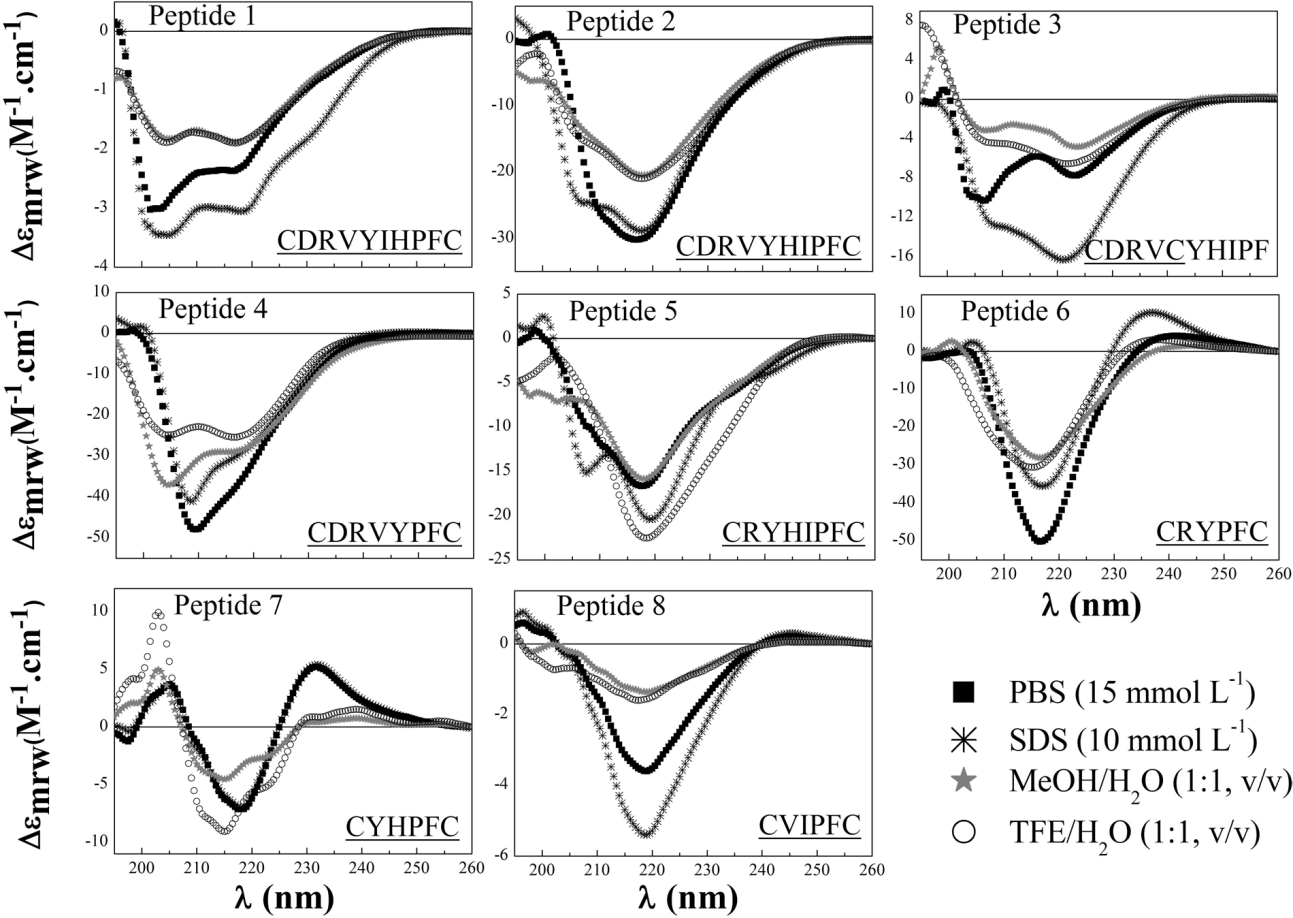
Circular dichroism spectra of constrained peptides. Spectra were recorded after four accumulations at 20 °C using a 0.5 mm path-length quartz cell between 260 nm and 195 nm at 50 nm/min with a band width of 0.5 nm. All peptides were analyzed in the following four solutions: 15 mM PBS, 10 mM SDS/PBS, 50% TFE/PBS, and 50% MeOH/PBS. The peptide concentration was approximately 10^−4^ mol L^−1^.

We then performed CD spectra experiments to determine the secondary structure of the peptides. The results obtained indicate that all peptides were unfolded and tended to adopt a random-coil conformation, except for peptides 6 and 7 that tended to form a β-turn^14^ structure in SDS solvent (Fig. 1).

### Effect of peptides against *Plasmodium gallinaceum* sporozoites during the pre-erythrocytic cycle of malaria

**Figure 2:**
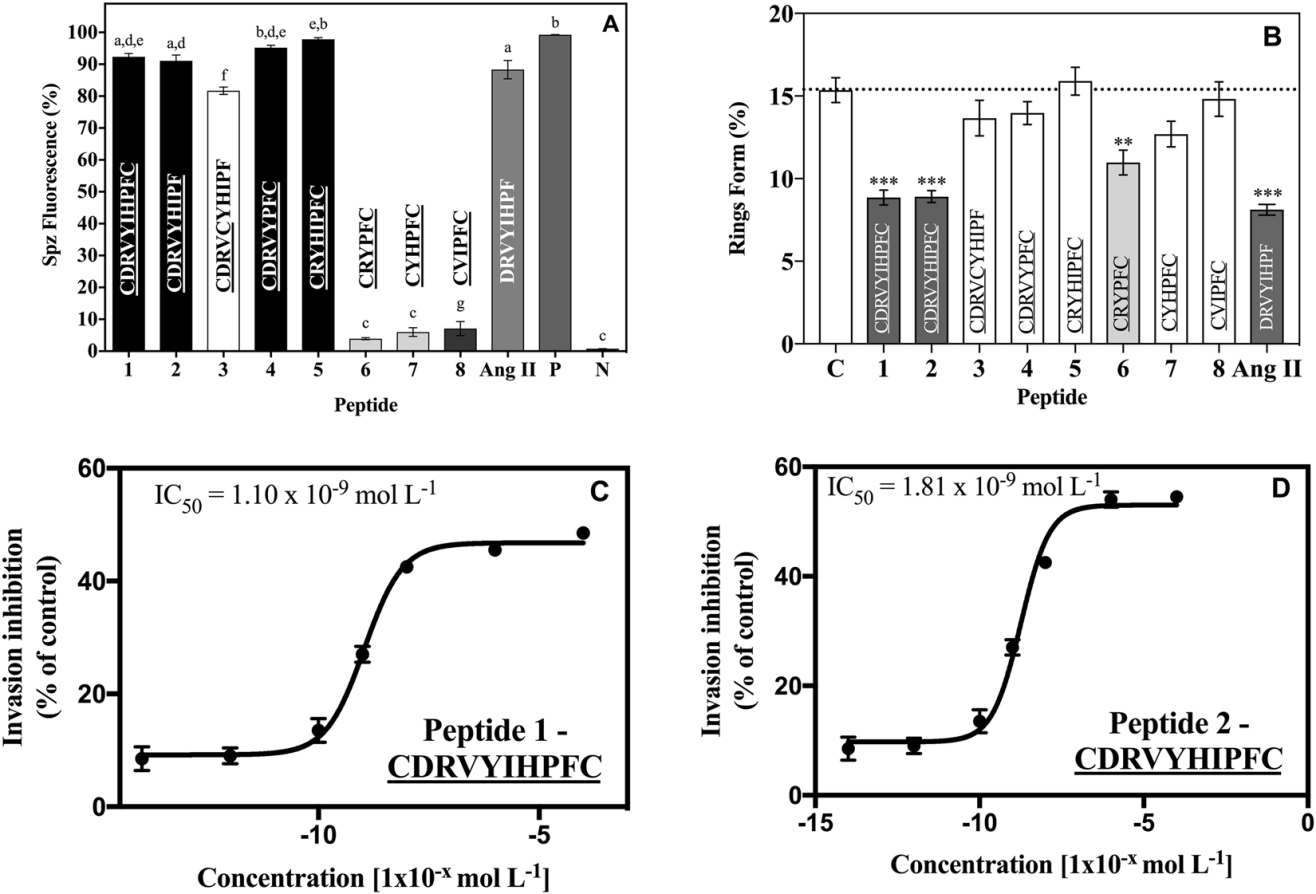
Antiplasmodial activity of designed synthetic peptides. (**A**) Effect of peptides on sporozoite membrane permeability expressed as % fluorescent mature sporozoites (mean ± standard deviation, n = 9). *Letters* indicate those results not significantly different from each other at p < 0.05 level. Positive control group (+): digitonin/PBS; negative control group (−): PBS. The most active peptides were 1, 2, 4 and 5 that presented 92, 91, 95 and 98% antiplasmodial activity, respectively. (**B**) Effect of peptides in new ring formation. The percentage of rings was determined after 24 h incubation of erythrocyte cultures infected with 2–3% schizonts in the absence (control) or presence of 10^−8^ mol L^−1^ of peptides. *Double asterisk* statistically significant compared with control value p < 0.01. *Triple asterisk* statistically significant compared with control value p < 0.001. *Dark grey shading* indicates that the result is statistically significant compared with control (mean ± standard deviation, *n* = 2). Data corresponding to ring form % in the presence of Ang II was retrieved from Saraiva *et al*.^15^. Ring formation inhibition (IC_50_ values) mediated by designed peptides 1 (**C**) and 2 (**D**). Peptides were diluted to seven concentrations [(10^−4^; 10^−6^; 10^−8^; 10^−9^; 10^−10^; 10^−12^; 10^−14^) mol L^−1^] leading to an inhibitory range between 6–54.5%. Data have been normalized due to differences between controls of each assay. The IC_50_ data were analyzed by GraphPad Prism analysis. Parameters: non-linear regression; log (inhibitor) vs response equation was chosen and least square (ordinary) fit method was applied (mean ± standard deviation, *n* = 2).

The effect of the peptides on sporozoites was determined *in vitro* by fluorescence microscopy. Briefly, the parasites were incubated for one hour in the presence of each peptide and the fluorophore propidium iodide, which stains dead and dying cells. The results are reported as the percent of fluorescent (i.e., inactivated) sporozoites. The analyses were carried out in triplicate, using three different mosquito batches (n = 9). The *in vitro* effect of peptides on *P. gallinaceum* sporozoites using our assay showed activity up to 98%, equipotent to the positive control digitonin, a surfactant that triggers uptake of propidium iodide into damaged/dead cells (Fig. 2A).

### Effect of peptides in the erythrocytic cycle of *Plasmodium falciparum*

The effect of peptides in the *P. falciparum* erythrocytic cycle was assayed *in vitro* against a synchronized culture in the schizont form of *P. falciparum*, maintaining 5% hematocrit and 2% parasitemia. Twenty-four hours after treatment, all peptides reduced new ring formation within erythrocytes at 10^−8^ mol L^−1^, which was described by Saraiva *et al*. as the ideal concentration for these inhibition assays^15^. The tests were carried out in duplicate and a total of ten experiments were performed (n = 10). Data were normalized due to differences between controls from each assay. It was observed that three analogs reduced the number of rings formed in the blood stage, but only analogs 1 and 2 presented inhibition >40%, when compared to the untreated controls (Fig. 2B).

The IC_50_ value represents the peptide concentration leading to a 50% reduction in the number of *P. falciparum* rings formed, when compared to untreated control cultures. The IC_50_ assays were performed using lead analogs 1 and 2, which presented >40% inhibition of ring form formation (Fig. 2C and D). Seven concentrations were tested with inhibitory activities that ranged from 8.5–54.5%.

### Hemolytic effect of peptides

**Figure 3:**
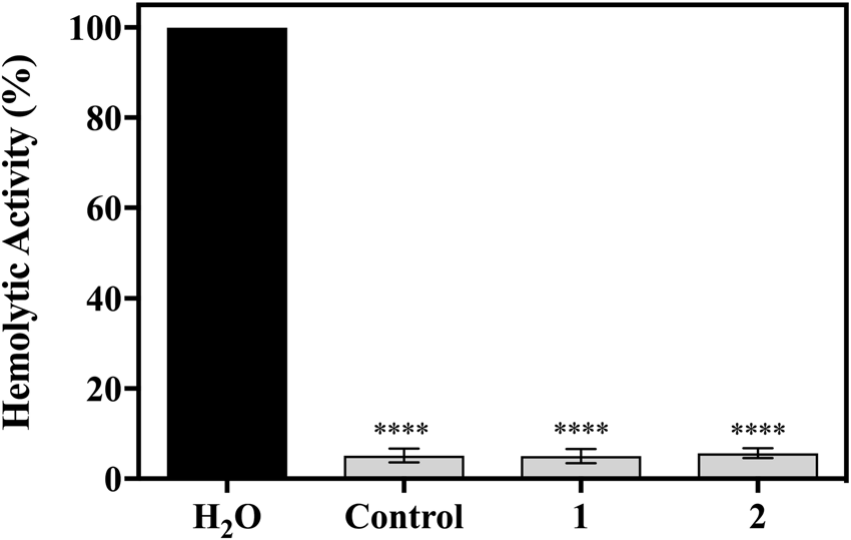
Hemolytic assay of human red blood cells. Red blood cells were treated with distilled water which causes lysis of red blood cells; uninfected erythrocytes were kept in the same conditions used in the invasion assay with a control and each peptide tested (10^−8^ mol L^−1^), at 37 °C for 24 h. After incubation, the supernatant was collected, clarified at 900 g/8 min and the hemoglobin content was detected in a spectrophotometer at 530 nm. **** denotes statistical significance of group compared with distilled water value p < 0.05. *Light grey shading* indicates that the result is not statistically significant compared with control (mean ± standard deviation, *n* = 3).

Assessing toxicity is a key requirement for translating novel compounds into the clinic. Therefore, we evaluated the hemolytic activity of Ang II peptide derivatives 1 and 2 towards erythrocyte human cells. Erythrocytes were seeded in 96-well plates in the absence (negative control) or presence of 10^−8^ mol L^−1^ of the analogs, as described in the Methods Section. The peptides did not present hemolytic effects (Fig. 3).

### Contractile response of peptides

**Figure 4:**
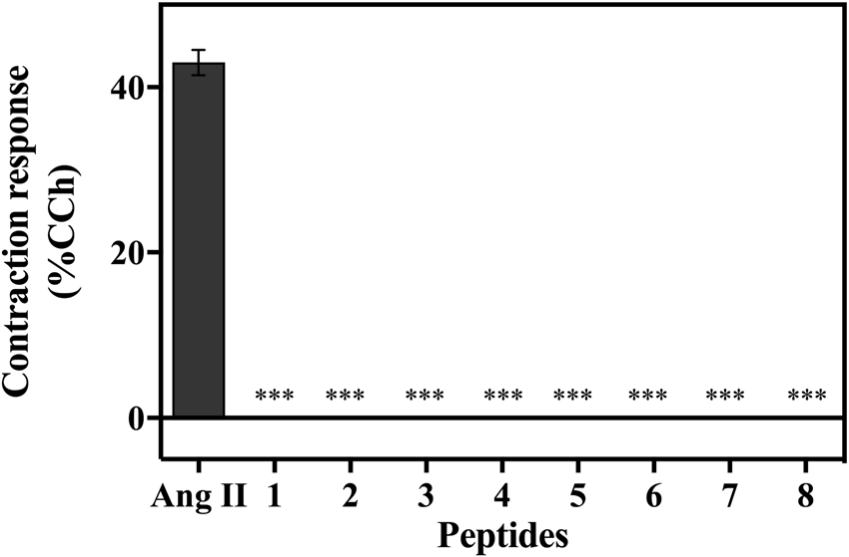
Effect of Ang II derived peptides in contractile responses by muscle tissue incubation compared to carbachol (CCh) activity. *Triple asterisk* statistically significant compared with control value p < 0.05 (mean ± standard deviation, *n* = 2).

Since one of the main limitations of Ang II as an antiplasmodial agent is its ability to increase blood pressure, here we tested the contractile response induced by our designer peptide analogs. These assays revealed that all peptides tested were inactive when compared to positive controls Ang II and carbachol (Fig. 4), therefore confirming the potential of lead peptides 1 and 2 as novel antimalarial therapies.

### Stability of peptides

**Figure 5:**
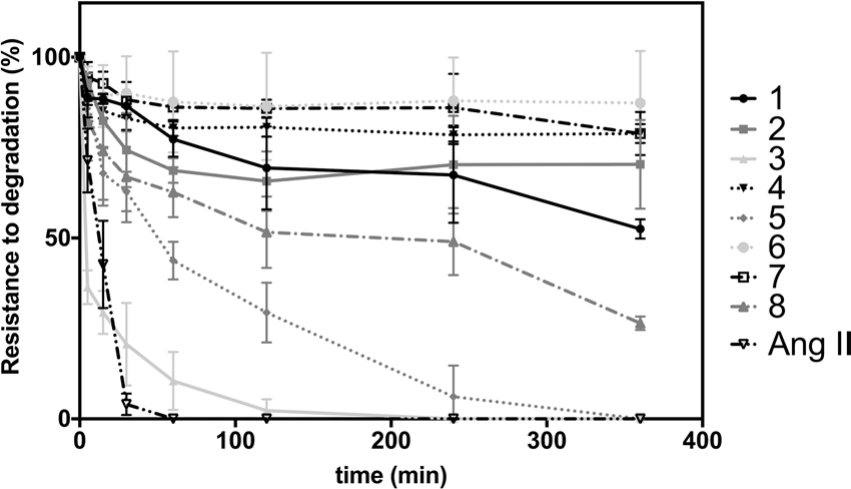
Stability of Ang II derivatives in human serum. Aliquots of peptides in the presence of human serum were taken over time (from 0 to 6 h) and were analyzed by LC-MS/ESI. The values shown correspond to % of area under the peak normalized according to the initial concentration of each peptide. All experiments were performed in duplicate.

A major concern for the translation of peptides into the clinic is their limited stability. Here we tested the resistance of our designer Ang II derivatives to degradation in human serum. These assays revealed that most of the constrained peptides tested were resistant to degradation in human serum (Fig. 5) even after a long time of exposure (6 hours). Therefore, these results confirm the expected increased stability of constrained peptides, and further highlight the antimalarial potential of lead peptides 1 and 2, which exhibited excellent stability in these assays.

## Discussion

As noted in previous work^7,8,16–18^, design of constrained peptides via S-S bonds contributes to the activity of peptides against *P. gallinaceum* sporozoites and leads to moderate activity (20–30%) against *P. falciparum* in the erythrocytic stage. This work presents the design of constrained peptides derived from the primary sequence of Ang II, which exhibit higher antiplasmodial activity than Ang II in two separate parasite models. Some of the designed peptides have a size advantage over their predecessors (~10 residues), as they are smaller (8 residues for peptide CRYHIPFC, which exhibited 98% antiplasmodial activity) and therefore more cost effective to manufacture as potential therapies. All of the constrained peptides presented high resistance to degradation in the presence of human serum proteases, except for the partially restricted analog 3 (Fig. 5), which presents exposed basic residues (i.e., preferential cleaving site for endopeptidases), and peptide 5 (Fig. 5), which has a positive charged group next to its N-terminus that likely exposes the Arg guanidinium group to proteases.

Manning *et al*. showed that even small peptide backbone conformational distortions can cause pronounced changes in CD signals, therefore demonstrating the suitability of CD to monitor conformational changes with high sensitivity^19^. Here, we used CD to: (A) verify the conformational tendency of constrained peptides cf. linear peptides^12^ and (B) propose a model for peptide-membrane interactions based on the conformational tendency adopted when peptides are restricted with disulfide bonds by using SDS micelles to mimic a biomembrane environment. Our findings revealed that (A): linear peptides^12^ adopted different β-turn conformations and restricted peptides tended to form random coil structures, except for peptides 6 and 7, which adopted β-turn conformations in SDS solvent (Figure 1)^14^. (B): peptides 1–5 and 8 tended to adopt random coiled conformations in SDS medium, a different behavior when compared to their linear analogs, which tended to adopt β-turn conformations.

Peptides were separated into three functional groups based on results obtained in the biological activity assays against *P. gallinaceum* sporozoites (Fig. 2A): group 1 – active peptides: with antiplasmodial activity >90%, which includes peptides 1, 2, 4 and 5; group 2 – peptide with moderate antiplasmodial activity (~82%), comprising peptide 3 (data is statistically significant compared with groups 1 and 3); and group 3 – non-active peptides: exhibiting antiplasmodial activity <15%, include peptides 6, 7 and 8.

Peptides 1 and 2 differ in residues Ile^5^ and His^6^. Analog 4 was designed via the removal of these two residues. Silva *et al*., studied linear peptides and observed that peptide DRVYHIPF had potent biological activity (94% against *P. gallinaceum* sporozoites). The sequence of peptide 1 is similar to that of Ang II, whereas peptide 2 presents a sequence that resembles Ang II with modifications in His and Ile residues (DRVYHIPF). Peptide 4, on the other hand, lacks Ile and His amino acid residues within its backbone. Furthermore, all peptides are restricted with a S-S bond, obtained through the insertion of two Cys residues in their extremities (both Nt and Ct). These results suggest that, for peptides 1, 2 and 4, the incorporation of S-S bonds did not interfere with the parasite-peptide interaction (i.e., antiplasmodial activity) when compared with Ang II or the linear counterpart of peptide 2, studied by Silva *et al*.^12^. We hypothesize that this may be due to the fact that restriction tends to promote rigidity on the molecule, therefore inducing intramolecular interactions between the peptide side chains and the potential for hydrogen bond formation with adjacent amino acids. In addition, disulfide bonds can lock peptide structure into its bioactive conformation, thereby enhancing selectivity and potency^17^.

Mutating Ile and His residues in peptide 5 to generate peptide 6 led to a substantial decrease in antiplasmodial activity: peptide 5 presented 98% antiplasmodial activity whereas peptide 6 showed 4% (Fig. 2). Depletion of antiplasmodial function upon mutation of Ile and His may be explained by: a) the presence of a disulfide bridge, which may lead to cation-π interactions between Tyr-His side chains, with possible hydrogen bonding^12^ b) interactions between residues that play an important role in the biological activity as the hydrophobic cluster formed by Tyr-His-Ile on hairpin structure of native Ang II, studied by Tzakos *et al*.^20^ c) the amount of amino acid residues, given that large S-S rings generally have higher biological activity^17^, and also observed in peptides 7 and 8.

Torres *et al*.^7^ studied Ang II analogs with S-S bonds and showed that restricted peptides presented antiplasmodial activity. Peptide sequence CDRVCYIHPF^7^ presented 76% (±3) antiplasmodial activity vs sporozoites of *P. gallinaceum*. Silva *et al*., studied different linear Ang II derivative peptides^12^ and observed that when His and Ile residues are inverted, the antiplasmodial activity increased cf. peptides with the native primary sequence. To verify this observation, here we inverted the position of Ile and His residues in peptide 3. The resulting peptide presented increased antiplasmodial activity [82% (±1)] compared to its analogs^7^.

Previous studies showed that Ang II and its analogs presented antiplasmodial activity in the erythrocytic stage as observed by Saraiva *et al*., using a *P. falciparum* erythrocytic cycle *in vitro* model^15^. The authors observed that Ang II reduced by 47% reinfection caused by ring forms. Silva and colleagues tested linear Ang II derivatives using the same model and observed that two short peptides were capable of reducing parasite reinfection >50%^12^. In this work, we tested designer peptides using the *P. falciparum* erythrocytic cycle and observed that two peptides (1 and 2) were able to reduce parasite reinfection >40% (Fig. 2B). Generally, larger restricted peptides (>5,000 Da) were more active than shorter (<500 Da) restricted peptides^13^. This is not entirely surprising as small peptide molecules are known to exhibit low target specificity which may interfere with their biological function^17^.

Only peptides 1 and 2 presented antiplasmodial activity in both models in addition to exhibiting resistance to degradation by human serum proteases (Fig. 5), thus subsequent studies focused on these two lead variants. The IC_50_ values of these peptides were determined *in vitro* (Fig. 2C,D) by testing seven concentrations resulting in 8.5–54.5% inhibition when using 1.10 × 10^−9^ mol L^−1^ of peptide 1 and 1.81 × 10^−9^ mol L^−1^ of peptide 2. Saraiva observed that Ang II at 10^−8^ mol L^−1^ of exerted its maximum effect at inhibiting parasite reinfection, as it reduced merozoite reinvasion by 47%^15^. To verify if peptides 1 and 2 had the same effect as Ang II, they were tested at 10^−8^ mol L^−1^ and led to parasitemia reductions of 42.5% for both peptides (Fig. 2B). The results showed that peptides 1 and 2 were able to reduce parasite reinfection to levels similar to Ang II^15^. In addition, we found that lead peptides 1 and 2 lacked hemolytic activity, as they induced <6% hemolysis of red blood cells (Fig. 3). Importantly, none of the peptides led to a significant contractile response (Fig. 4), which is an important limitation of Ang II.

Overall, we have designed novel restricted peptides that are mostly resistant to degradation by proteases and that exhibit potent antiplasmodial activity *in vitro* against *Plasmodium gallinaceum* sporozoites. Four peptides presented >90% activity against *Plasmodium*. Two of them presented inhibitory activity that was comparable to that of the positive control digitonin, which promotes 100% lysis of parasite membranes. Peptides 4 and 5 constitute promising candidates for the treatment of malaria in its pre-erythrocytic stage. Restricted peptides presented potential antiplasmodial activity *in vitro* against erythrocyte invasion by *Plasmodium falciparum*. Peptides 1, 2 and 6 were able to reduce reinfection by the parasite, whereas peptides 1 and 2 presented equipotent activity in both models and were equipotent to Ang II. Peptides 1 and 2 are promising pre-erythrocytic and erythrocytic drug candidates that show potential in the prevention of malaria. On the other hand, peptide 6 was the only one that presented activity exclusively against erythrocyte invasion by *P. falciparum*. Although its biological activity was not equipotent to Ang II, the selectivity demonstrated by this peptide makes it an interesting template sequence for the design of peptides that selectively target specific stages of the *Plasmodium* life cycle. Vasoconstriction suppression was achieved in all cases as none of the peptides led to contractile responses and the restriction design strategy proved to lead to resistance to degradation by proteases for most peptides, except for the ones that presented basic residues exposed (Fig. 5), thus reinforcing the promise of these molecules as novel drugs.

In the sporozoitic stage of the *P. gallinaceum* cycle (Fig. 2A), our peptides may interact with *P. gallinaceum* cells by binding and destabilizing the membrane of *P. gallinaceum* sporozoites, as previously reported (Maciel *et al*., PLoS One, 2008). On the other hand, in the merozoitic stage of the *P. falciparum* cycle (Fig. 2B), our peptides may operate via a distinct mechanism, which is currently under investigation.

In conclusion, we present novel stable and nontoxic Ang II synthetic stable peptide derivatives with antiplasmodial function that represent promising agents for the prevention and treatment of malaria.

## Methods

### Peptide Synthesis

Peptides were synthesized by manual solid-phase synthesis; we leveraged 9-fluorenylmethyloxycarbonyl (Fmoc)^21^ using Wang^22^ resin (aapptech, USA) with ~0.5 mmol g^−1^ substitution degree. A treatment with 20% 4-methylpiperidine (4-MePip) in dimethylformamide (DMF) for 40 min was applied to unprotect Fmoc-Amino acids (NovaBiochem) residues. Couplings were carried out using 2,5-fold molar excess of the Fmoc-protected amino acid, activated by N,N′-diisopropylcarbodiimide (DIC)/Hydroxybenzotriazole (HOBt), for 2 hours at room temperature, in Dichloromethane (DCM)/DMF (1:1, v/v) and monitored by the colorimetric Kaiser test^23^. Each step was followed by a washing procedure with DMF, methanol and DCM to alternately change the degree of resin swelling, favoring the elimination of excess reagents and byproducts^24^.

To cleave the peptide from the resin, dry-protected peptidyl-resin was exposed to trifluoroacetic acid (TFA)/water/anisole (95:2.5:2.5, v/v/v) for two hours at room temperature. The crude deprotected peptides were precipitated with anhydrous diethyl ether, filtered from the ether-soluble products, extracted from resin with 60% acetonitrile (ACN) in water and lyophilized.

The S-S bonds were formed by first solubilizing the crude peptides (1 g L^−1^) in an 80% acetic acid solution containing 0.04 mol L^−1^ iodine^25^. After 40 min, the reaction was extracted with water and diethyl ether. After that, the diethyl ether was evaporated, and the resulting solution was lyophilized.

The crude peptides were purified by preparative reverse-phase high-performance liquid chromatography (RP-HPLC) in 0.1% TFA/H_2_O (solvent A) and 0.1% TFA/60% ACN/H_2_O (solvent B) on a Delta Prep 600 (Waters Associates system, Milford, MA, USA). Briefly, the peptides were loaded onto a Phenomenex C_18_ (21.2 × 250 mm, 15 µm particles, 300 Å pores) column at a flow rate of 10.0 mL min^−1^ and eluted using a linear gradient (0.33% solvent B/min slope) of 0.1% TFA − 60% ACN/H_2_O with detection at 220 nm. Selected fractions containing the purified peptides were pooled and lyophilized. Purified peptides were characterized by liquid chromatography electrospray ionization mass spectrometry (LC/ESI-MS) (Table 1). LC/ESI-MS data were obtained on a Model ZMD mass spectrometer (Micromass, Milford, MA, USA) coupled to a Model 2690 HPLC system (Waters Alliance, Milford, MA, USA) using a Phenomenex Gemini C_18_ column (2.0 × 150 mm, 3.0 µm particles, 110 Å pores). Solvent A was 0.1% TFA in water, and solvent B gradient was performed over 30 min, and peptides were detected at 220 nm. Mass measurements were performed in a positive mode with the following conditions: mass range between 500 and 2000 m/z, nitrogen gas flow rate at 4.1 L h^−1^, capillary voltage at 2.3 kV, cone voltage at 32 V, extractor at 400 °C, ion energy at 1.0 V and a multiplier at 800 V.

### Circular Dichroism Spectroscopy

The peptides were measured in four solutions: 15 mmol L^−1^ PBS (pH = 7.4), 10 mmol L^−1^ SDS in PBS, 50% TFE in PBS, and 50% MeOH in PBS. The peptide concentration was 10^−4^ mol L^−1^. (FAR-UV) CD (190–260 nm) spectra were recorded at room temperature. It was using a 0.5 mm path-length quartz cell in a Jasco J815 spectropolarimeter (Tokyo, Japan). Accumulations of four analyses were recorded for the spectra data. The scan rate was 50 nm min^−1^ with band width of 0.5 nm. The CD spectra for the SDS, TFE and MeOH solutions were subtracted, and to minimize background effects a Fourier transform filter (FFT) was applied.

### Mosquito rearing and maintenance of the parasite life cycle

*Aedes aegypti* RED strain was used in experiments due to their hypersensitivity to *P. gallinaceum* parasite^26^. Mosquitoes were reared using standard laboratory procedures^27^. An aliquot of frozen chicken blood infected with the *P. gallinaceum* strain 8 A was obtained from A Krettli (René Rachou Institute of Research, FIOCRUZ, MG, Brazil). This sample was used to inoculate and establish initial infections in chickens. All subsequent infections of chickens and mosquitoes were accomplished by feeding the mosquitoes on the chickens.

### Effect of peptides on salivary gland-derived *Plasmodium gallinaceum* sporozoites

Nine thousand *P. gallinaceum* mature sporozoites were pulled from the salivary glands of *A. aegypti* and incubated in 50 μL of PBS solution, with 40 μmol L^–1^ digitonin (positive control), 60 μmol L^–1^ peptides or negative control (PBS solution), at 37 °C for one hour. Cell membrane integrity was then observed using a Carl Zeiss inverted fluorescence microscope (model Observer Axio Vision A.1) coupled to an image capture Zeiss AxioCam HR digital camera (1,300 × 1,030 pixels resolution and 8-bit quantization) after addition of 1 μL of the propidium iodide aqueous solution (200 μmol L^–1^) in 5 μL of total solution volume. Images were obtained using a 40X objective lens and a green filter effect in red. The spectral range was set with the excitation at 538 nm within the visible spectrum in order to produce orange-red fluorescence centered at 619 nm, which was processed using the Axio 4.7 software.

### Plasmodium falciparum *in vitro* assay

The erythrocytic cycle of *Plasmodium falciparum* was maintained *in vitro* by culturing W2 strains in 1640 medium, supplemented with A-type human blood and serum, as described by Saraiva *et al*.^15^ The parasite first samples were donated by Dr. Mariano Zalis from the Laboratory of Infectology and Parasitology, Hospital Universitário Clementino Fraga Filho, Universidade Federal do Rio de Janeiro, Rio de Janeiro, Brazil.

### Erythrocytic invasion assessment by *Plasmodium falciparum* assay

To synchronize the parasite cultures into the ring stage, we used a sorbitol solution (5%) according to Lambros and Vanderberg^28^. The resistant ring forms were maintained in culture as described above. Erythrocytes infected by parasite mature forms at 2–3% parasitemia and 5% hematocrit were kept in the presence or absence of 10^−8^ mol L^−1^ synthetic peptides, for 24 h. Invasion was evaluated by the appearance of new ring forms. The invasion percentage was determined by optical microscopy (1000x magnification), which represents the total number of cells infected by rings in 100 erythrocytes in at least ten random microscopic fields. The invasion assay was carried out using two different cell suspensions and generated equivalent results.

### IC_50_ value determination

A dose-response relationship assay was carried out for 24 h with concentrations ranging from 10^−14^ to 10^−4^ mol L^−1^ of each peptide in order to determine the IC_50_ values for peptides 1 and 2 for suppression of erythrocytic invasion by *P. falciparum*. The IC_50_ values were calculated using GraphPad Prism Software version 5.00 for Windows (GraphPad, San Diego, California, USA). Parameters: Nonlinear Regression; log (inhibitor) vs. response equation was chosen at least square (ordinary) fit method was applied.

### Hemolytic effect of peptides

The hemolytic effect of analogs 1 and 2 was determined in uninfected erythrocytes. Cells were maintained in the same culture conditions in the presence of 10^−8^ mol L^−1^ of peptides for 24 h. Erythrocytes were centrifuged at 900 g for 8 min. The free hemoglobin in the supernatant was monitored by spectrophotometer at 550 nm. Untreated cells were used as negative control to rule out nonspecific hemolysis. The hemolytic activity was expressed as a percentage of the control. This positive control (100% hemolysis) was prepared by treating the erythrocytes with distilled water.

### Contractile response assays

The bioassay experiment was carried out in duplicate and generated equivalent results. Experiments were carried out using C57BL/6 J mice from the Centro de Desenvolvimento de Modelos Experimentais da Universidade Federal de São Paulo (CEDEME-UNIFESP).

Stomach fundus was isolated from each mouse, divided into two strips along the longitudinal muscle, and mounted into 5 mL organ baths containing modified Krebs-Ringer solution [144 mmol L^–1^ NaCl, 5 mmol L^–1^ KCl, 1.1 mmol L^–1^ MgSO_4_, 25 mmol L^–1^ NaHCO_3_, 1.1 mmol L^–1^ NaH_2_PO_4_, 1.25 mmol L^–1^ CaCl_2,_ and 5.5 mmol L^–1^ glucose at 37 °C (pH 7.4)] and were continuously carboxygenated (95% O_2_/5% CO_2_)^29^. Contractile responses to the stimulations with Ang II and analogs 1 and 2 (10^–8^ mol L^–1^) were measured with a TRI201 tension transducer (PanLab) through an amplifier (Powerlab 4/30). Data were collected through Labchart Pro V7 software. The resting tension was maintained at 0.5 g and the tissues were left to equilibrate for 90 min. The bath solution was frequently changed. The maximal effect was obtained by comparing contractile responses induced by carbachol (10^–8^ mol L^–1^).

### Stability on human serum

The Ang II derivatives resistance to degradation assay was carried out in human serum of healthy donors. A 20 μL aliquot of a 10 mg mL^−1^ peptide solution was added to 980 μL of a 25% fresh serum solution in water. The new solution was incubated at 37 °C and 50 μL were taken in 0, 5, 15, 30, 60, 120, 240 and 360 min. Serum protein were precipitated by adding 5 μL of TFA and after 10 min of incubation the solutions were centrifuged (300 g) for 5 min. The supernatant (20 μL) was injected in a LC-MS/ESI system. Absorbance in 0 min was measured and the percentage of reminiscent peptide was calculated by comparing peak areas of the next aliquots to the concentration of the first measurement.

### Statistical analysis

The experiments were performed in independent cell suspensions. The data were analyzed by one-way analysis of variance using treatments as factors. The significance of the differences was verified by the Bonferroni adjustment. Statistical analysis was performed using absolute values; the data are expressed as the mean ± standard deviation: n = 3. All of them were considered statistically significant compared with the control value (or water distilled in erythrocytic haemolysis assay), if *p* < 0.05. GraphPad Prism version 5.00 for Windows was used (GraphPad Software, San Diego, California, USA).

### Ethics statement

#### Plasmodium falciparum and erythrocyte hemolysis assays

The collection of human blood samples for this study was conducted according to protocol 074/10 and approved by the Research Ethics Committee of the Hospital Universitário Clementino Fraga Filho at the Universidade Federal do Rio de Janeiro. Informed consent was obtained to enable collection of human blood samples for this study.

#### Plasmodium gallinaceum

The collection of *Gallus gallus domesticus* blood samples for this study was conducted according to the current guidelines for the care and use of laboratory animals as well as the ethical guidelines for investigations, and these experiments were preapproved by the Animal Care Committee of the Universidade de São Paulo, number 133.

### Contractile response assay

The pharmacological experiment followed the current guidelines for the care and use of laboratory animals as well as the ethical guidelines for investigations, and these experiments were preapproved by the Animal Care Committee of the Universidade Federal de São Paulo, number 2013/479357.
